# Pregnancy and lambing rates following direct transfer of vitrified embryos in field ewes of Bangladesh

**DOI:** 10.5455/javar.2025.l968

**Published:** 2025-09-30

**Authors:** Md. Monirul Islam, Mohammad Rafiqul Islam Talukder, Nazmun Naher, Pankaj Kumar Jha, Mohammad Musharraf Uddin Bhuiyan, Farida Yeasmin Bari

**Affiliations:** 1 Department of Surgery and Obstetrics, Faculty of Veterinary Science, Bangladesh Agricultural University, Mymensingh, Bangladesh; 2 Department of Livestock Services, Dhaka, Bangladesh; 3 Bangladesh National Zoo, Dhaka, Bangladesh; 4 Nepal Agricultural Research Council (NARC), Kathmandu, Nepal

**Keywords:** Sheep embryos, vitrification, direct transfer, field ewes, pregnancy rate, lambing rate

## Abstract

**Objective:**

Embryo vitrification facilitates multiple ovulation and embryo transfer (MOET) application in the sheep industry through the storage and transfer of genetically superior embryos. This study assessed the survival rate of vitrified embryos following direct transfer under field conditions.

**Materials and Methods:**

Thirty-five donors and 46 recipient ewes were synchronized for estrus using two injections of Cloprostenol. Superovulation was induced with 25 mg porcine follicle-stimulating hormone per donor twice daily for 4 days. Recipients were treated with 250 IU of pregnant mare serum gonadotrophin during the second injection of cloprostenol to ensure ovulation. Estrus donors were mated with rams. Embryos were collected on day 6 post-mating using a modified inguinal laparotomy and graded. Grade 1 embryos were vitrified in a medium containing tissue culture medium 199, 10% ethylene glycol, 10% dimethyl sulfoxide, and 0.5M sucrose and stored in liquid nitrogen. Following thawing, embryos were directly transferred to recipients through an open-pulled straw following an inguinal laparotomy. Sixteen recipients were treated with 20 µg Gonadorelin immediately after embryo transfer.

**Results:**

Onset and duration of estrus in donor and recipient ewes were 30.2 ± 0.8, 27.9 ± 0.6, and 33.7 ± 0.4, 27.50 ± 0.42 h, respectively. Corpora lutea number and recovered embryos/donor were 8.47 ± 0.68 and 6.93 ± 0.57, respectively. 85.7% of donors responded to superovulation treatment, and the embryo recovery rate was 82.5%. Grade 1 embryos per donor (5.5 ± 0.8) were significantly higher (*p* < 0.05) than all other grades. Pregnancy rates in recipients treated with Gonadotrophin-releasing hormone (*GnRH*) and without *GnRH* treatment were 62.5% and 56.6%, respectively. The respective lambing rates were 80% and 76.5%.

**Conclusion:**

These findings indicate the potential on-farm application of direct transfer of vitrified embryos in facilitating a MOET program for genetic improvement of sheep in Bangladesh.

## Introduction

The development of the livestock sector has been a significant priority in Bangladesh for the last 2 or 3 decades. Sheep are one of the important livestock species in Bangladesh, especially in the areas where people suffer from extreme poverty, for example, Char Island. The ultra-poor people of Char Island are mainly sheep breeders. In these areas, sheep could provide a major contribution to food security, income generation, and poverty alleviation. However, poor genetic merit and the lack of proper breeding management systems are the major impediments to the advancement of the sheep farming industry in these low-income areas [1]. The multiple ovulation and embryo transfer (MOET) technique is used worldwide for speeding up rates of genetic gain, producing a large number of high genetic merit livestock, and reducing the generation interval [2]. Vitrification is a cryopreservation technique that employs a rapid cooling of a liquid-based solution that, in turn, transforms the liquid into a glass. It can be achieved by using high cooling rates and a high concentration of cryoprotective agent. Embryo vitriﬁcation for storage using the open pulled straw (OPS) technique and their subsequent direct transfer into recipient ewes offers a real possibility to reduce the cost of embryo transfer [3,4] with potential applications under ﬁeld conditions [5].

Several prior studies on the vitrification of lamb embryos have been conducted abroad [4,5]. However, the poor genetic merits of local sheep in Bangladesh need the speedup of genetic gain through the female line (MOET technique) for producing a large number of quality lambs in this economically deprived country. Lamb production following fresh embryo transfer using the MOET technique was successful in Bangladesh [6–8]. Direct transfer of vitrified embryos with lamb production was conducted at the research station [9]. It was urgent to introduce the technology at the field level to (i) make the MOET technique cost-effective, (ii) see the success of the technique in field ewes, and (iii) acceptability of the technique by farmers. Hence, a pilot study was designed to observe the lambing outcomes following vitrified embryos in local sheep of Bangladesh under field conditions. The objective of this study was to assess pregnancy and lambing rates following direct transfer of vitrified embryos into recipient ewes under field conditions. Administration of exogenous Gonadotrophin-releasing hormone (*GnRH*) to pregnant animals during various times of gestation has been shown to increase pregnancy rates [10,11]. Consequently, the study also investigated the effects of *GnRH* administration on pregnancy and lambing rates following vitrified embryo transfer in local ewes under field conditions.

## Material and Methods

### Ethical approval

The research design was based on guidance provided by the UK Animals (Scientific Procedures) Act, 1986, and the EU Directive 2010/63/EU, and approved by the Animal Experimental Ethics Committee (AEEC), Department of Surgery and Obstetrics, Bangladesh Agricultural University, Mymensingh (Ref. no. AEEC/DSO–BAU/02/2015).

### Study area

The study was carried out at Sheep Research Farm of the Department of Surgery and Obstetrics (DSO), Faculty of Veterinary Science, Bangladesh Agricultural University, Mymensingh–2202, and Kandapara village of Fulpur Upazilla, Mymensingh district.

### Selection and management of the ewes

A total of 35 nonpregnant, healthy high-genetic-merit ewes, 5–6 months old, from the University Sheep Research Farm, were selected as donors. For recipient ewe selection, a workshop was conducted with 30 sheep farmers from the Fulpur Upazila having 5–15 sheep, and 46 ewes were selected as recipients from 12 of these farms.

### Oestrus synchronization and detection in donor and recipient ewes

The donor ewes were synchronized for estrus by injecting two doses of 175 µg cloprostenol (Ovuprost^®^ Bayer, New Zealand) intramuscularly 9 days apart. Estrus was confirmed by observing behavioral and clinical signs of synchronized ewes at 2 h intervals (30 min at each observation) starting from 24 h following the 2nd injection of cloprostenol, using a vasectomized ram. The recipient ewes were synchronized using the same method as the donor except that they were treated with 250 IU pregnant mare serum gonadotrophin (PMSG) (Folligon^®^, Intervet, Boxmeer, Netherlands) at the time of the 2nd injection of cloprostenol to ensure ovulation.

### Superovulation of donor ewes

Donor ewes were injected intramuscularly with 25 mg of porcine follicle-stimulating hormone (*pFSH*) (Folltropin-v^®^, Bioniche Animal Health Canada Inc.) at 12 h intervals starting from day 10 of the 2nd injection of cloprostenol within the schedule for 4 consecutive days.

### Hand mating of donor ewes

Donor ewes were hand-mated to fertile rams of high vigor to achieve fertilization of induced ova following confirmation of estrus.

### Preparation of media

Flushing medium was prepared using tissue culture medium (TCM) solution with 10% fetal bovine serum (FBS). For holding medium (HM), 20% FBS was added to the TCM. For equilibration solution (ES), 10% ethylene glycol (EG) and 10% dimethyl sulfoxide (DMSO) were used. The vitrification solution contained 20% EG, 20% DMSO, and 0.5M sucrose. A warming solution was prepared using TCM solution, with 10% FBS and 0.5M sucrose.

***Embryo collection***

On 6.5–7 days after mating, embryos were collected from donor ewes by modified inguinal laparotomy (inguinal cut combined with non-suture of peritoneum). Ewes were premedicated using 0.22 mg/kg body weight (BW) Xylazine (Xylazine^®^, Indian Immunological Ltd, Hyderabad, India) given intramuscularly and anesthetized with Ketamine (G-ketamine^®^, Gonoshasthya) at 2 mg/kg BW intravenously. Anesthetized ewes were placed on a cradle in dorsal recumbence at a 45° angle in the head-down position. A 4–5 cm paramidline skin incision was made (4 cm cranial and 8 cm lateral to the udder) following disinfection with povidine iodine (Povisep^®^, Jayson Pharmaceuticals Ltd). Following exteriorization of the reproductive tract, an 8-G two-way Foley catheter was introduced into the lumen of the uterine horn through a hole made by a blunt needle in a position just below the uterine bifurcation. The number of ovulations was determined by counting the corpus luteum (CL) number on the ovary. Flushing medium was flushed through the horn using a 19-G blunt needle inserted into the tip of the horn, and the flushing medium containing embryos was collected via the Foley catheter into a pre-warmed sterile Falcon tube. The uterine horn was replaced into the abdominal cavity following careful removal of the Foley catheter after deflation of the balloon. The same procedure was repeated for the other uterine horn. The internal wound was then sutured using catgut, and the skin wound was closed with silk thread.

### Embryo searching and grading

The collected medium containing the embryos was kept at 37°C for 10 min to settle down. The collection medium was then poured into a pre-warmed graduated petri dish, and the embryos were searched for using a Stereomicroscope (Olympus, SZX2–TR30, Tokyo, Japan). Embryos were evaluated and graded as per the guidelines provided by the International Embryo Transfer Society.

### Embryo vitrification and storage

The grade 1 embryos in pairs were first equilibrated in HM for 10 min and then second equilibrated in the ES for 3 to 5 min, followed by vitrification solution for >40 sec. The embryos were then loaded into OPS and accumulated in a goblet and placed into liquid nitrogen for storage in a Cryocan.

### Direct transfer of vitrified embryos

The embryos were warmed up in the warming solution for 6 sec at 37°C. A Tom Cat catheter attached to an air-filled 1 ml syringe having a hole in its barrel was inserted into the OPS containing the embryos. The thawed embryos were then transferred into the uterine horn of recipients on day 6.5 or 7 post-estrus by inguinal laparotomy using the same surgical approach described for the donor ewe. Following laparotomy, the tip of the uterine horn ipsilateral to the ovary containing a greater number of CL was exteriorized and punctured with a blunt 19-G needle. The tip of the OPS was inserted through the punctured wound of the horn, and the whole content of the OPS containing the two embryos was transferred into the uterine horn by depressing the plunger of the syringe. The abdominal wound was closed in the same manner as mentioned for donor ewes.

### GnRH treatment for the recipients

Of the 46 recipients used, 16 were also treated with an intramuscular injection of 20 µg Gonadorelin (0.2 ml Fertagyl^®^, Intervet, Boxmeer, Netherlands) immediately after embryo transfer.

### Pregnancy diagnosis

Pregnancy was confirmed by transabdominal ultrasonography (DRAMINSKI^®^ Animal Profi, Poland) 35–40 days after transfer of vitrified embryos.

### Statistical analysis

Statistical analysis was done using Statistical Package for the Social Sciences Version 20.0. The values were expressed as mean ± SEM. The comparison of embryo grading and pregnancy rate was performed using chi-square analysis, with *p* < 0.05 considered statistically significant.

## Results

### Oestrus synchronization in donor and recipient ewes

The mean values for the onset and duration of estrus are shown in Table 1. The onset of estrus varied from 24 to 48 h in donors and 30 to 40 h in recipient ewes following the 2nd cloprostenol injection. The duration of estrus varied from 24 to 36 h in both donor and recipient ewes. The onset of estrus was significantly (*p* < 0.01) earlier in donor ewes (30.17 ± 0.80 h) compared to recipient ewes (33.73 ± 0.41 h). However, the duration of estrus was similar in both donor and recipient ewes (27.89 ± 0.58 h *vs.* 27.50 ± 0.42 h).

### Superovulation and embryo yield

Superovulatory response (CL number), number of embryos, and embryo recovery rate of donor ewes are shown in Table 2. The numbers of CL and recovered embryos varied from 3 to 18 and 3 to 16, respectively.

**Table 1.** Synchronization of estrus in donor and recipient ewes.

Group	Onset of estrus (h)	Duration of estrus (h)
Donor (*n* = 35)	30.17 ± 0.80^b^	27.89 ± 0.58
Recipient (*n* = 46)	33.73 ± 0.41^a^	27.50 ± 0.42
Significance level	**	NS

The values in the same column with different superscripts differed significantly (*p* < 0.01). NS = not significant.

**Highly significant (*p* < 0.01).

**Table 2.** Superovulation with *pFSH* and embryo yield (mean ± SEM).

Parameters	Donors (*n* = 35)
Donor ewes super ovulating (%)	85.71 (30/35)
Number of CL/donors	8.47 ± 0.68
Number of embryos/donors	6.93 ± 0.57
Embryo recovery rate (%)	82.49 ± 2.17

**Table 3.** Total numbers, mean (± SEM) per donor and percentage of grade 1, 2, 3, and 4 embryos.

Embryo grading	No. of embryos	Mean ± SEM	Percentage (%)
Grade 1	140	5.5 ± 0.8a	67.6
Grade 2	28	0.93 ± 0.26b	13.5
Grade 3	21	0.70 ± 0.21b	10.1
Grade 4	18	0.60 ± 0.15b	8.7
Significance level		**	

The values in the same column with different superscripts differed significantly (*p* < 0.01).

**denotes statistical differences among rows at α = 0.05.

**Table 4.** Pregnancy and lambing rates following transfer of vitrified embryos into recipient ewes.

Parameters	With *GnRH* treatment	Without *GnRH* treatment	Significance level
Pregnancy rate (%)	62.5 (10/16)	56.6 (17/30)	NS
Lambing rate (%)	80 (8/10)	76.5 (13/17)	

NS = not significant.

### Quality of embryos

The mean values of embryo quality are presented in Table 3. The number and percentage of grade 1 embryos (5.5 ± 0.8 and 67.6 %) were significantly higher (*p *< 0.01) than other grades.

### Effects of GnRH on pregnancy and lambing rates following vitrified embryo transfer

Pregnancy and lambing rates within the *GnRH*-treated group and non-treated group are shown in Table 4. Pregnancy rates in *GnRH*-treated and non-treated recipient ewes were 62.5% and 56.6%. Lambing rates were 80% and 76.5%, respectively.

## Discussion

Vitrification of sheep embryos facilitates the storage and transportation of high genetic quality embryos, contributes to reduced health risks compared with other breed improvement strategies, and makes it relatively easy to apply MOET to indigenous flocks under field conditions, speeding up the rate of genetic improvement of traits of high economic importance. Our study is the first in Bangladesh to observe the survival rate of vitrified embryos following direct transfer in indigenous ewes under field conditions. The increased number of quality lambs with vitrified embryos acquainted the farmers with the manipulative reproductive technology for increased lamb production. This knowledge will facilitate the buildup of the sheep industry in Bangladesh.

Mean values of onset and duration of estrus following the second cloprostenol injection in donor and recipient ewes are very similar to those of Roy et al. [12]. In small ruminants, estrus synchronization is achieved either by reducing the length of the luteal phase of the estrous cycle with Prostaglandin F2α or by extending the cycle with exogenous progesterone [13,14]. Zohara [7] showed that two injections given 9 to 11 days apart resulted in estrus in all ewes with increased fertility and therefore was the method of choice used in this study.

Donor ewes showed estrus significantly earlier than recipient ewes. This could be due to the higher degree of follicle stimulation arising from high doses of follicle stimulating hormone compared with PMSG, as well as the different environments where donor and recipient ewes were maintained. The selected quality donor ewes were maintained in the research station with care and management different from the field environment.

However, the duration of estrus was similar for both donors and recipients. Superovulation is the principal conventional approach to utilize female genetic potential effectively and rapidly by producing a higher number of quality embryos from valuable high genetic merit females within an MOET program [15]. 85.71% of donors superovulated, with a mean of 8.47 ± 0.68 CL per donor. These findings are consistent with those of Zohara et al. [16]. The number of embryos recovered per donor ewe and the embryo recovery rate were 6.93% ± 0.57% and 82.49% ± 2.17%, respectively. The findings are like those reported by Munoz et al. [17]. The proportion of grade 1 embryos was significantly higher (*p* < 0.01) than all the other grades, and this agrees with the result of Ghosh et al. [9]. In this study, a modified inguinal laparotomy approach for embryo recovery was used, which is inexpensive and easily applicable in field conditions. The technique also helps to prevent abdominal herniation, adhesion, and other postoperative complications [6].

Previous research has shown that vitrified embryo transfer using both direct and indirect methods showed similar results in terms of pregnancy rate (60%–75%) and lambing rate (55%–75%) [18].

The indirect technique has some limitations, as it involves a complex process of embryo thawing and obligatory embryo evaluation under the microscope before transfer, which is both expensive and time-consuming. By comparison, embryo vitriﬁcation for storage using the OPS technique followed by direct transfer significantly reduces the cost of embryo transfer, providing potential applications under field conditions typical of the more primitive farming situations to improve genetic quality within their sheep industry [4].

In the present study, pregnancy rates and lambing rates were not significantly influenced by *GnRH* treatment of recipient ewes. They were similar to international standards and comparable with those of Khan et al. [19]. Post-mating *GnRH* injection on day 0 and 9 has previously been shown to enhance pregnancy rate and litter size in ewes, and this may be due to *GnRH* increasing progesterone production from the CL through stimulating release of luteinizing hormone from the anterior pituitary [20]. Although not significantly higher, however, both pregnancy and lambing rates tended to be increased following *GnRH* injection following the transfer of vitrified embryos in the current study, and this lack of statistical significance may be attributable to the relatively small group sizes involved.

## Conclusions

The poor genetic merit of local sheep needs breed upgradation to increase and improve lamb production in Bangladesh. The supply of vitrified embryos to farmers’ sheep on demand will enhance the genetic improvement program. In the present study, the pregnancy and lambing rates following the direct transfer of vitrified embryos in field ewes are excellent, making the MOET technique cost-effective to implement. The development of a feasible MOET protocol, vitrification of embryos, and a pilot study in the field will encourage sheep farmers to rear sheep using manipulated reproductive technology to fulfill their dream.

## List of abbreviations

AEEC, Animal Experimental Ethics Committee; BAU, Bangladesh Agricultural University; BW, body weight; cm, centimeter; DMSO, dimethyl sulfoxide; DSO, Department of Surgery and Obstetrics; EG, ethylene Glycol; ES, equilibrium solution; FBS, fetal bovine serum; G, gauge; *GnRH*, Gonadotrophin releasing hormone; HM, holding medium; h, hours; IU, International Unit; kg, kilogram; mg, milligram; ml, milliliter; MOET, Multiple Ovulation and Embryo Transfer; NATP–BARC, National Agricultural Technology Program – Bangladesh Agricultural Research Council; ºC, degree celsius; OPS, open pulled straw; *pFSH*, porcine follicle stimulating hormone; PMSG, pregnant mare serum fonadotrophin;TCM, tissue culture medium; µg, microgram; µl, microliter.
